# Assessing the Performance of ChatGPT in Answering Patients’ Questions Regarding Congenital Bicuspid Aortic Valve

**DOI:** 10.7759/cureus.72293

**Published:** 2024-10-24

**Authors:** Mousumi Barua

**Affiliations:** 1 Internal Medicine, School of Public Health and Health Professions, University at Buffalo, Buffalo, USA

**Keywords:** artificial intelligence (ai), bicuspid aortic valve, chatgpt, ethical concerns, healthcare, medical education, public health

## Abstract

Aim: Artificial intelligence (AI) models, such as ChatGPT, are widely being used in academia as well as by the common public. In the field of medicine, the information obtained by the professionals as well as by the patients from the AI tools has significant advantages while at the same time posing valid concerns regarding the validity and adequacy of information regarding healthcare delivery and utilization. Therefore, it is important to vet these AI tools through the prism of practicing physicians.

Methods: To demonstrate the immense utility as well as potential concerns of using ChatGPT to gather medical information, a set of questions were posed to the chatbot regarding a hypothetical patient with a congenital bicuspid aortic valve (BAV), and the answers were recorded and reviewed based on three criteria: (i) readability/technicality; (ii) adequacy/completeness; and (iii) accuracy/authenticity.

Results: While the ChatGPT provided detailed information about clinical pictures, treatment, and outcomes regarding BAV, the information was generic and brief, and the utility was limited due to a lack of specific information based on an individual patient’s clinical status. The authenticity of the information could not be verified due to a lack of citations. Further, human aspects that would normally emerge in nuanced doctor-patient communication were missing in the ChatGPT output.

Conclusion: Although the performance of AI in medical care is expected to grow, imperfections and ethical concerns may remain a huge challenge in utilizing information from the chatbots alone without adequate communications with health providers, despite having numerous advantages of this technology to society in many walks of human life.

## Introduction

As technology and societal infrastructure improve, so does the availability of medical information to the general public. With the growing access to the internet, patients have significantly fewer barriers to acquiring adequate and often redundant medical knowledge [1]. The advent of search engines, such as Google, Bing, Yahoo, and many others, provides the internet users of the modern generation access to vast amounts of information [2]. To many older medical professionals, this was considered a profound change in how patients can access medical information and gain some understanding of their symptoms and conditions, and perhaps something to be wary of. Technological progress brings along with it more resources to be made available to the patient population in particular [3].

Within the last few years, there has been a sudden boom of interest and involvement in artificial intelligence, commonly referred to as AI, in every sector of technology and our daily lives [4]. A specific type of AI that produces text, what most people think of as a “chatbot,” is what is known as a large language model or LLM. These large language models are built on a predictive framework through analysis of preexisting information to procedurally generate text. ChatGPT, probably the most popular and well-known application of LLMs, was recently developed by “OpenAI” (https://openai.com/), a startup company that was founded in September of 2022. Nowadays, ChatGPT is being used by students, software engineers, and the common public for a plethora of uses [4]. An important concern for healthcare providers to be cognizant of is the misuse of these LLMs and the potential for misinformation for their patients [5]. As the utility of AI models, such as ChatGPT, is becoming ubiquitous in academia [6], industry [7], and medicine [8,9], including by the general public, an important concern is the ethical issues surrounding its use and misuse not only in healthcare delivery by professionals but also in its utilization by patients [10].

Given the above context, it is becoming increasingly important to understand the performance of the chatbot with regard to its potential to produce relevant and useful information that professionals and the public are seeking from it. As an example for this exercise, I chose the bicuspid aortic valve (BAV), a congenital medical condition in which the baby is born with only two leaflets in the aortic valve instead of the typical three [11]. The main reason for choosing this topic is twofold: (i) I had a keen interest in cardiology and came across several cases of BAV while working in a cardiology unit for over a year, and this can facilitate me in framing questions for the chatbot, and (ii) BAV is recognized as the most common congenital heart defect and represents up to 6.5 million individuals in the United States [12]. Epidemiological studies have reported that the incidence of isolated BAV cases in the general population is about 1% with a prevalence rate of 0.5-0.8% in healthy school children and young adults, while the prevalence rate in patients with aortic coarctation is as high as 50-85% (cited from Spaziani et al. [13]). BAV poses a significant health burden to both patients and the health system, as morbidity and mortality rates are higher than the combination of all other congenital heart defects [11,14]. Therefore, a focus on accessing medical information regarding BAV might benefit many people, including caregivers and healthcare providers.

A BAV is the most common congenital heart defect in which the BAV contains two cusps (commissures) instead of the usual three [11]. BAV is three to four times more common in males than in females, which may be linked to a reduced dosage of X chromosome-related genes as males have a single X chromosome [15]. While the cardiac complications associated with BAV usually develop in adulthood, 12-15% of pediatric patients require intervention during their childhood and adolescence [16]. BAV has many subtypes and a wide spectrum of clinical presentations, ranging from silent malformation to severe and even fatal cardiac events [17]. BAV is associated with an aortopathy manifested by dilatation of the ascending thoracic aorta, a condition requiring periodic monitoring of aortic diameters, elective prophylactic surgical aortic repair, and the occurrence of aortic dissection or rupture [18]. Most BAV patients at some stage require aortic valve replacement (AVR) and aortic surgery [19]. The mean age of BAV patients undergoing the surgery was 45.3 years, with a 91.2% survival rate 10 years after surgery [20]. While BAV lends itself to a significant burden on both the patients and the health systems, scientific understanding of its pathophysiology, risk factors, and genetics is rather poor [11]. Recent studies have identified specific gene variants associated with BAV [21,22], suggesting that BAV is a complex disorder with a significant genetic influence [22].

## Materials and methods

To demonstrate the effectiveness and possible limitations of ChatGPT in educating the patient population, I used a set of questions from a hypothetical patient with BAV and collected the response provided by ChatGPT (model version: gpt-4o-2024-05-13) so that I could evaluate the effectiveness of these responses and how well the technology can inform the patients regarding their medical conditions, treatment options, and outcomes [23]. In this context, as a trained clinician with a medical background, I framed the questions from the point of view of a typical BAV patient after consulting with two cardiologists for input to finalize the question. The hypothetical patient in the current study, as extrapolated from the data presented in a cohort study of BAV in children and young adults [24], is a 20-year-old college student who was found to have a fusion of right- and left-coronary leaﬂets. The presenting complaints included chest pain, fatigue, shortness of breath, and feelings of dizziness or lightheadedness over the past two months, and the symptoms reportedly worsened during the last few days. A heart murmur was recorded during clinical examination, and BAV was diagnosed by echocardiography, which showed mild aortic stenosis and regurgitation. The patient was referred for a balloon angioplasty as an initial valve intervention, although a surgical aortic valvuloplasty or valve replacement would have been considered in other severe and chronic cases of BAV. Finally, I queried the ChatGPT with five selected questions that were deemed from the hypothetical BAV patient, and then I reviewed the answers delivered by the chatbot.

The main hypothesis was that the ChatGPT would generate relevant and technical information for the questions posed by the patient, but the content would not be sufficient and easy for the patients to understand various complex and heterogeneous clinical presentations of BAV. Having a background and experience in cardiology, the author of this study framed the following five questions from the point of view of a typical patient or patient’s caregiver: (i) What is a bicuspid aortic valve?; (ii) Why do I have a bicuspid aortic valve?; (iii) What are the causes of concern if I have a bicuspid aortic valve?; (iv) What are the available treatments for the bicuspid aortic valve?; and (v) How about the outcome (prognosis) for the bicuspid aortic valve? These five questions were chosen for this study because, in the author’s own clinical experience, these were among the most commonly asked questions posed by the patients and caregivers to the doctors about their pediatric patients with BAV. Further, these questions were acknowledged by two cardiologists as some of the most frequent questions that come up from the BAV patients and their caregivers during clinical interactions.

A free version of ChatGPT (model version: gpt-4o-2024-05-13), which was based on GPT-4, was used for testing the hypothesis by entering each question at the search portal of the website https://chatgpt.com/, although there are several models currently available and being updated (see https://platform.openai.com/docs/models). Registration of a user account at the website was required to use the tool by the general public. The response for each question was copied and tabulated (see Table 1). The content was reviewed based on three criteria: (i) readability/technicality; (ii) adequacy/completeness; and (iii) accuracy/authenticity. Relying mainly on the clinical experience of the author, an informal, unstructured qualitative analysis was done to test the hypothesis and formulate recommendations regarding the potential use of ChatGPT for patients with BAV to understand their medical condition and potential interventions (see Table 2).

**Table 1:** Questions from the user and responses from the ChatGPT

Question from hypothetical patient	Response of ChatGPT about bicuspid aortic valve
What is a bicuspid aortic valve?	A bicuspid aortic valve (BAV) is a congenital heart defect where the aortic valve has only two leaflets (or cusps) instead of the normal three. This condition can affect the way blood flows from the heart into the aorta and onward to the rest of the body. Here are some key points about BAV: 1. Anatomy and Function: The aortic valve is located between the left ventricle of the heart and the aorta, the main artery that carries oxygen-rich blood to the body. Normally, the aortic valve has three cusps (tricuspid). In BAV, only two cusps are present. 2. Prevalence: BAV is one of the most common congenital heart defects, occurring in about 1-2% of the population. 3. Symptoms and Complications: Many people with BAV are asymptomatic and may not know they have it. Complications can include aortic valve stenosis (narrowing of the valve), aortic valve regurgitation (leakage of the valve), and an increased risk of aortic aneurysm (dilatation of the aorta). Symptoms, when present, may include chest pain, shortness of breath, fatigue, dizziness, and palpitations. 4. Diagnosis: BAV can be diagnosed using imaging techniques such as echocardiography, MRI, or CT scans. 5. Treatment: Monitoring: Regular check-ups and imaging to monitor the condition. Medication: To manage symptoms and prevent complications. Surgery: Valve repair or replacement may be necessary if significant stenosis or regurgitation occurs or if an aortic aneurysm develops. 6. Prognosis: Many individuals with BAV live normal, healthy lives, especially with regular medical follow-up and management of any complications. Early detection and appropriate management are crucial for preventing serious complications. Overall, while a bicuspid aortic valve is a significant congenital condition, advancements in medical monitoring and treatment have greatly improved outcomes for those affected.
Why do I have a bicuspid aortic valve?	A bicuspid aortic valve (BAV) is a congenital heart defect where the aortic valve has only two cusps (or leaflets) instead of the normal three. Here are some key points to understand why you might have a bicuspid aortic valve: Genetics: BAV often runs in families, suggesting a genetic component. If you have a family history of BAV or other congenital heart defects, you may have inherited a gene that contributed to the development of your condition. Developmental Factors: The aortic valve forms during the first few weeks of fetal development. Sometimes, the developmental processes involved in forming the valve can be disrupted, leading to a bicuspid valve instead of a tricuspid valve. Incidence: BAV is one of the most common congenital heart defects, affecting about 1-2% of the population. It is more common in males than in females. Associated Conditions: BAV can be associated with other cardiovascular conditions, such as aortic aneurysms, coarctation of the aorta, or other valvular problems. The exact cause of BAV can sometimes be linked to broader developmental or genetic conditions affecting the heart and blood vessels. Sporadic Cases: In some instances, BAV occurs sporadically without a clear genetic or familial pattern. This means that while there may be genetic predispositions, environmental factors or random developmental variations could also play a role. If you have been diagnosed with a bicuspid aortic valve, it's important to have regular follow-ups with a cardiologist to monitor the condition, as BAV can lead to complications like aortic stenosis (narrowing of the valve) or aortic insufficiency (leakage of the valve). Your cardiologist can provide personalized advice and management based on your specific situation.
What are the causes of concern if I have a bicuspid aortic valve?	Having a bicuspid aortic valve (BAV) means that your aortic valve has only two leaflets (or cusps) instead of the normal three. This congenital heart defect can lead to several concerns and potential complications: Aortic Stenosis: The valve can become narrowed (stenotic) over time, leading to restricted blood flow from the heart to the aorta and the rest of the body. Symptoms may include chest pain, fatigue, shortness of breath, and fainting. Aortic Regurgitation: The valve may not close properly, causing blood to leak back into the left ventricle from the aorta (regurgitation). This can lead to left ventricular dilation and heart failure if severe and untreated. Aortic Aneurysm: The aorta may become enlarged (aneurysm) due to the altered blood flow dynamics and structural abnormalities associated with BAV. This poses a risk of aortic dissection or rupture, which are life-threatening emergencies. Infective Endocarditis: BAV increases the risk of infective endocarditis, an infection of the heart valves. This risk is due to the abnormal structure of the valve, making it more susceptible to bacterial infection. Left Ventricular Hypertrophy: Increased workload on the heart due to stenosis or regurgitation can cause thickening of the left ventricular muscle (hypertrophy), potentially leading to heart failure over time. Arrhythmias: Patients with BAV may be at higher risk for developing heart rhythm abnormalities (arrhythmias), which can affect the heart’s efficiency and lead to symptoms like palpitations, dizziness, or fainting. Family History and Genetic Concerns: BAV can run in families, indicating a possible genetic component. Relatives of individuals with BAV might be at increased risk and may need screening. Monitoring and Management: Individuals with BAV require regular follow-up with a cardiologist. Monitoring typically includes Echocardiograms: To assess valve function, aortic size, and heart muscle condition. CT or MRI: To monitor the size of the aorta and detect aneurysms. Electrocardiograms (ECG): To check for arrhythmias. Management and Monitoring Regular monitoring through echocardiograms or other imaging techniques is essential to keep track of any changes in the valve or aorta. Management strategies might include: Medical Management: Medications to control blood pressure, reduce the risk of endocarditis, and manage symptoms. Surgical Intervention: Valve repair or replacement surgery may be necessary, particularly if stenosis, regurgitation, or aortic aneurysm becomes severe. Lifestyle Adjustments: Regular exercise and a heart-healthy diet can help manage symptoms and reduce the risk of complications. Close follow-up with a cardiologist who specializes in congenital heart defects is essential for optimal management of a bicuspid aortic valve.
What are the available treatments for the bicuspid aortic valve?	Treatments for a bicuspid aortic valve (BAV) depend on the severity of the condition and any associated complications. Here are the main options: Monitoring: If the BAV is not causing significant issues, regular monitoring through echocardiograms may be recommended to track its function over time. Medications: Patients may be prescribed medications to manage symptoms or prevent complications, such as blood pressure medications (like ACE inhibitors) if there's associated aortic regurgitation or hypertension. Aortic Valve Repair or Replacement: Valve Repair: In some cases, the valve can be repaired, though this is less common with BAV compared to other valve types. Valve Replacement: If the valve is severely narrowed (aortic stenosis) or leaking (aortic regurgitation), surgical replacement with either a mechanical or biological valve may be necessary. Transcatheter Aortic Valve Replacement (TAVR): This less invasive procedure may be an option for some patients, particularly older individuals or those with other health issues. Surgical Aortic Root Replacement: If there is dilation of the aorta, this procedure may be needed to replace the aorta and the valve. Lifestyle Modifications: Patients are often advised to adopt a heart-healthy lifestyle, including regular exercise, a balanced diet, and monitoring cholesterol and blood pressure. Regular follow-ups with a cardiologist are crucial to determine the appropriate treatment plan based on the individual’s condition.
How about the outcome (prognosis) for the bicuspid aortic valve?	The prognosis for individuals with a bicuspid aortic valve can vary significantly based on several factors, including the presence of symptoms, the degree of aortic stenosis or regurgitation, and the overall health of the individual. Mild Cases: Many people with a bicuspid aortic valve may not experience significant problems and can live normal, healthy lives. Regular monitoring is typically recommended. Moderate to Severe Cases: Over time, the valve can become stenotic (narrowed) or regurgitant (leaky), which may lead to symptoms like shortness of breath, chest pain, or fatigue. In these cases, the prognosis can worsen without intervention. Surgical Intervention: If surgery (valve repair or replacement) is needed, the prognosis improves significantly. Many patients experience good outcomes post-surgery, although regular follow-up is necessary. Associated Risks: Those with a bicuspid aortic valve may have an increased risk of aortic dilation or aneurysm, so ongoing monitoring of the aorta is essential. Overall, with appropriate management and regular follow-ups, many individuals with a bicuspid aortic valve can lead fulfilling lives.

**Table 2:** Qualitative evaluation of the ChatGPT responses based on three criteria (readability/technicality, adequacy/completeness, and accuracy/authenticity). Scores represent the quality of the answer ranging from 1 to 5 1: very poor; 2: poor; 3: acceptable; 4: good; 5: excellent

Question	Readability/technicality	Adequacy/completeness	Accuracy/authenticity
What is a bicuspid aortic valve?	Score: 3/5. While some technical terms are defined (stenosis - narrowing of the valve; regurgitation - leakage of the valve; aneurysm - dilatation of the aorta), some of the terms (e.g., leaflets, cusps) needed definitions and/or illustrations.	Score: 3/5. While the question is aimed at a definition and/or description of BAV, the ChatGPT answer briefly covered multiple aspects, rendering commission errors. Each aspect covered in the answer is not adequate to get a clear understanding of BAV. A physician may have provided adequate information according to the needs of specific patients.	Score: 3/5. Some of the sentences are “overly” positive clinical pictures and outcomes. Examples: “Many people with BAV are asymptomatic and may not know they have it” and “Many individuals with BAV live normal, healthy lives, especially with regular medical follow-up and management of any complications”. The reported clinical outcomes in percentage would be better. No references/sources are included in the answer.
Why do I have a bicuspid aortic valve?	Score: 3/5. Major etiological factors are briefly summarized in moderately technical language along with definitions in easy language.	Score: 3/5. The description of etiological factors is very short and inadequate. A physician may have offered a context-specific interpretation for this question. The last two advisory sentences are not relevant to the question.	Score: 3/5. The information about the lack of scientific understanding regarding the etiology and pathophysiology of BAV is absent in the ChatGPT answer, and this could mislead the reader to think that the etiology of BAV is fully known. No references/sources are included in the answer.
What are the causes of concern if I have a bicuspid aortic valve?	Score: 3/5. The complications associated with BAV are briefly mentioned in moderately technical language along with simple definitions. However, some of the terms may need additional interpretation and/or illustrations (e.g., left ventricular dilation) for easy understanding.	Score: 3/5. The description of complications associated with BAV is very short and inadequate. A physician may have offered context-specific feedback for this question. Additional topics such as monitoring, management, and lifestyle adjustments are beyond the focus of the question.	Score: 4/5. Various complications (concerns) associated with BAV are accurately listed, albeit briefly. As there are no references/sources included in the answer, verification or additional probe is not possible.
What are the available treatments for the bicuspid aortic valve?	Score: 4/5. Six different treatment options are listed and briefly explained in simple terms.	Score: 3/5. While the major treatment options have been briefly explained, the information appears to be grossly inadequate. A medical professional may have provided additional and relevant information depending on the patient’s specific condition.	Score: 4/5. All major treatment options, as currently available, have been briefly mentioned. While the information seems to be accurate, the absence of references or sources for the content in the answer makes verification of the information very difficult.
How about the outcome (prognosis) for the bicuspid aortic valve?	Score: 3/5. The prognosis for BAV has been listed based on severity and intervention in simple, clear terms, although some technical terms lack definitions (e.g., aortic dilation, aneurysm).	Score: 3/5. While the prognosis for BAV has been summarized based on severity and treatment options, the content is too brief. Additional information on clinical outcomes in terms of percentage for different treatment methods may have been more useful.	Score: 3/5. Most of the information contained in the answer is found to be accurate. However, there is a scope for misunderstanding some statements without accurately specifying the proportion of cases. Example: “Many people with a bicuspid aortic valve may not experience significant problems and can live normal, healthy lives.” As with other questions, sources or references for the content are missing.

## Results

The questions supplied by the author and the answers by the chatbot are listed in Table 1. The text output by the ChatGPT has been reproduced verbatim in the table. Notably, the text output did not contain any citation sources or references.

Table 2 lists the qualitative analysis based on three criteria: (i) readability/technicality; (ii) adequacy/completeness; and (iii) accuracy/authenticity. Overall, the ChatGPT provided only a generic answer regardless of a patient's age, gender, and clinical status, while a physician or cardiologist could provide personalized information, advice, and management based on the patient’s specific situation.

## Discussion

BAV is the most common and complex congenital heart disease, affecting 1-2% of the population [25]. Clinical sequelae and complications of BAV include infectious endocarditis, aortic dilatation, aortic dissection and coarctation, aortic valve stenosis, and aortic valve regurgitation [26]. Over 50% of patients with BAV would require cardiac surgery during their lifetime, and severe aortic valve stenosis is associated with an annual mortality of 25% if left untreated [25]. BAV carries more morbidity and mortality rates than the combination of all other congenital heart disorders, thus imposing a significant burden on patients, families, and the health system [14]. However, the etiology and pathogenesis of BAV are poorly understood [27]. The current study evaluated the performance of ChatGPT for specific questions from the point of view of patients with BAV (Table 1) using a qualitative analysis based on three criteria (Table 2).

The three criteria used to assess the answers generated by the ChatGPT for the selected five questions from the point of view of a typical BAV patient are (i) readability/technicality; (ii) adequacy/completeness; and (iii) accuracy/authenticity. Overall, the chatbot has provided answers in the form of a summary for each question with necessary subheadings and additional suggestions or instructions. This is in clear contrast to the results from a search engine (e.g., Google, Yahoo, Bing, etc.), which provides numerous links for different websites and resources related to the search term, often leaving the user with information overload, unlike the ChatGPT, which provides a compiled brief summary that the user can quickly go through. For each question, the quality score assigned to each criterion achieved the level of “acceptable” and “good.” The scores were neither to the “poor” end nor achieved “excellent,” owing to the inherent design of the ChatGPT with its ability to provide the most necessary information but within a brief format at the cost of quality, details, and context. Further, none of the answers produced by the chatbot contained the source or reference material for the content for further citation or verification.

Judging by the criteria of readability/technicality, the answer becomes more useful and user-friendly if the content is explained in both technical and simple terms since the chatbot does not know the specific user or whether the question comes from an expert professional (e.g., physicians, scientists, etc.) or an individual from the general public (patients, students, etc.). As the chatbot is blind to the history and/or status of the individual user, the readability/technicality of the content is not expected to be specific to the user's specific needs. As with other categories, the chatbot performed well in the range of acceptable to good for the readability/technicality criterion, although it did not achieve an excellent score as there were technical terms lacking definitions and/or explanations. In terms of adequacy/completeness, while most of the answers contain major aspects of the topic to be addressed, the scores were in the medium range as the answers were found to be inadequate for each question. However, recognizing the fact the answer is only generic and not based on an individual user/patient, it is appreciable that the chatbot adds a final remark at the end of the answer, such as “Your cardiologist can provide personalized advice and management based on your specific situation” and “Regular follow-ups with a cardiologist are crucial to determining the appropriate treatment plan based on the individual’s condition.” Likewise, the accuracy/authenticity score did not achieve an excellent score mainly because of missing sources or references for the content presented in the answers.

Further, it was also observed that ChatGPT, despite its limitations in the current format, provided valuable information in short summaries. In addition, although the answers from the ChatGPT primarily lacked important and nuanced aspects of the doctor-patient interaction, it could suggest a kind of professional advice such as “close follow-up with a cardiologist who specializes in congenital heart defects is essential for optimal management of a bicuspid aortic valve," “your cardiologist can provide personalized advice and management based on your specific situation," and “regular follow-ups with a cardiologist are crucial to determine the appropriate treatment plan based on the individual’s condition.” Although ChatGPT brings up relevant information about BAV and its possible complications and available treatments in snippets, the source and the authenticity of the information remain unverified. Hence, the technology, in its current level of sophistication, poses a challenge in terms of all three categories, such as readability/technicality, adequacy/completeness, and accuracy/authenticity. By virtue of these limitations, the ChatGPT in its current format is described as a “jack of all trades, master of none” [28]. Further, the perception of ChatGPT by people and professionals is paradoxical in nature: while the technology has significant utility in compiling and generating a vast amount of information instantly, it raises great concerns about its application in healthcare settings [29] as the field of medicine critically involves people’s lives and well-being. These concerns are probably centered around potential misinformation in the output generated by the ChatGPT as well as possible misuse of information by the patients.

There could be a variety of ethical considerations for using ChatGPT in healthcare [5,30]. As explained in detail by Wang et al. [30], these ethical issues in using AI and its content in the healthcare setting include (i) legal issues related to holding responsibility for the content used, privacy issues, licensing, and regulations; (ii) humanistic issues such as ambiguity concerning “compassionate care” as suggested by the AI, less importance to the physician-patient relationship due to easy availability of information from the AI, and integrity issues surrounding the AI-generated content; (iii) algorithmic issues including potential bias in the content generated by the AI algorithms, assuming responsibility and accountability to the content generated by the AI, concerns regarding lack of transparency and explainability of the content, and validation and evaluation of its content regarding accuracy, reliability, and effectiveness; and (iv) information-related issues such as data bias in the training data of AI models, validity or reliability of the AI-generated content, and effectiveness of the AI models in producing “accurate” and verifiable information. On the other hand, despite these ethical concerns, utilizing AI technology holds great potential for global public health [31] in terms of clinical applications [23,32] as well as medical research [33]. Specifically, the AI models are predicted to become an important asset to medical education [34,35], medical writing [36], and patient care related to diagnosis and treatment [37,38].

Any tool, whether it be a search engine, website, or artificial intelligence, has the potential to be used either to help the patients gather necessary information about their medical condition or to misinform or impede the adequate provision of healthcare to the patient. Similarly, a scalpel is only as useful as how it is utilized. If used properly in a surgical context by someone who has training and expertise, it becomes a tool to heal; if in the hands of someone untrained, it presents a clear danger. In the same vein, AI technology can also be misused. While AI tools such as ChatGPT offer convenience to patients by allowing them to collect a simple, clear-cut answer to their question without having to read an article or conduct detailed research, this convenience comes at the cost of accountability and reliability [39]. With how simple it is to ask a question and receive an answer, the patient loses the nuance of the implications of their symptoms and their condition, which normally comes out in doctor-patient communications [9]. However, despite these limitations and challenges, patients and the public alike may immensely benefit from AI models like ChatGPT to get much-needed information so quickly and easily. Based on this limited exercise, I would concur with the proposal from the professionals that policies and guidelines concerning the use of AI, especially in healthcare, need to be developed by various stakeholders to mitigate legal and ethical issues [10,29].

Limitations

It must be acknowledged that the current study has many limitations, some of which could be addressed by future studies. First of all, the input questions for the ChatGPT regarding BAV (Table 1) were framed by the author based on her clinical experience and the feedback from two physicians, rather than from an empirically validated set of questions. Second, the criteria formulated for the qualitative analysis as well as the assignment of scores for the output produced by the ChatGPT (Table 2) may not be devoid of subjective bias. Structured sets of clinical information from the published literature on BAV to algorithmically evaluate the quality and accuracy of the ChatGPT output may have been a preferred method, while this may entail a separate project that is beyond the scope of the current study. Third, only a small set of five questions were queried to the ChatGPT, which may be perceived as inadequate to cover various aspects of BAV. Fourth, just one of the many versions of the ChatGPT (GPT-4o-2024-05-13) was used in the current study to evaluate the performance, while the possibility remains that the other models, including the paid or commercial model versions, may have performed differently. Fifth, while the ChatGPT is the most popular chatbot used by a vast majority of people currently, the framed questions were not tested on the other AI-based language models (e.g., Microsoft’s Copilot, Google’s Gemini, etc.) to permit a comparison of performances across these chatbots. Finally, it may be feasible to compare the outputs between a chatbot such as ChatGPT and that from a search engine (e.g., Google, Yahoo, Ping, etc.), although this aim may require a massive and labor-intensive project to differentiate the voluminous output from the search engine from a small summary output from the chatbot, which is beyond the scope of the current study. We suggest that future studies should try to address some of these limitations.

## Conclusions

The current study evaluated the performance of ChatGPT using a qualitative analysis of the answers provided by the chatbot to specific questions from the point of view of a typical patient. Overall, the performance of ChatGPT was found to be moderate, as the quality of the answers from ChatGPT ranged from acceptable to good, given that there were limitations in the content of the answers. With the ease of receiving quick answers to their burning questions, the patients may often lose the nuance of the implications of their symptoms and their condition, which is an essential part of doctor-patient communication. Although the need for AI in medical care is expected to grow exponentially, imperfections and ethical concerns will remain a huge challenge in utilizing information from chatbots alone without adequate communications with health providers. However, despite these limitations and challenges, patients and the public alike may immensely benefit from AI models like ChatGPT to get needed information quickly and easily. As with other information available on the internet, caution must be exercised regarding the authenticity, adequacy, and source of the information, including those obtained from AI models such as ChatGPT.
